# Synthesis of mesoscopic particles of multi-component rare earth permanent magnet compounds

**DOI:** 10.1080/14686996.2020.1862630

**Published:** 2021-01-22

**Authors:** T. Thuy Trinh, Jungryang Kim, Ryota Sato, Kenshi Matsumoto, Toshiharu Teranishi

**Affiliations:** Institute for Chemical Research, Kyoto University, Uji, Kyoto, Japan

**Keywords:** 102 Porous / Nanoporous / Nanostructured materials, 103 Composites, 106 Metallic materials, 203 Magnetics / Spintronics / Superconductors, 301 Chemical syntheses / processing

## Abstract

Multielement rare earth (R)–transition metal (T) intermetallics are arguably the next generation of high-performance permanent magnetic materials for future applications in energy-saving and renewable energy technologies. Pseudobinary Sm_2_Fe_17_N_3_ and (R,Zr)(Fe,Co,Ti)_12_ (R = Nd, Sm) compounds have the highest potential to meet current demands for rare-earth-element-lean permanent magnets (PMs) with ultra-large energy product and operating temperatures up to 200°C. However, the synthesis of these materials, especially in the mesoscopic scale for maximizing the maximum energy product (${\left(BH\right)}_{max}$), remains a great challenge. Nonequilibrium processes are apparently used to overcome the phase-stabilization challenge in preparing the R–T intermetallics but have limited control of the material’s microstructure. More radical bottom-up nanoparticle approaches based on chemical synthesis have also been explored, owing to their potential to achieve the desired composition, structure, size, and shape. While a great achievement has been made for the Sm_2_Fe_17_N_3_, progress in the synthesis of (R,Zr)(Fe,Co,Ti)_12_ magnetic mesoscopic particles (MMPs) and R–T/T exchange-coupled nanocomposites (NCMs) with substantial coercivity ($H_{c}$) and remanence ($M_{r})$, respectively, remains marginal.

## Graphical abstract

**Figure:**
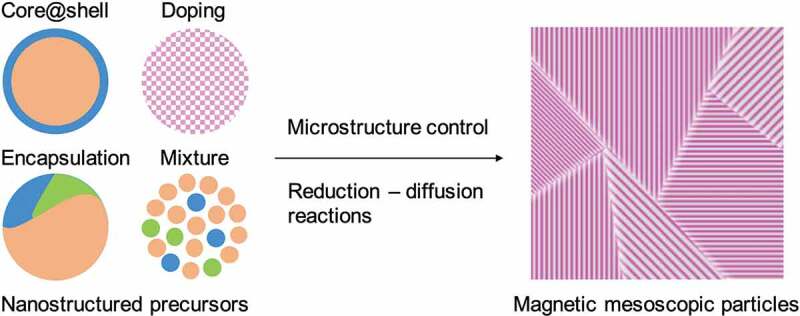

## 1. Introduction

**Figure 1.**
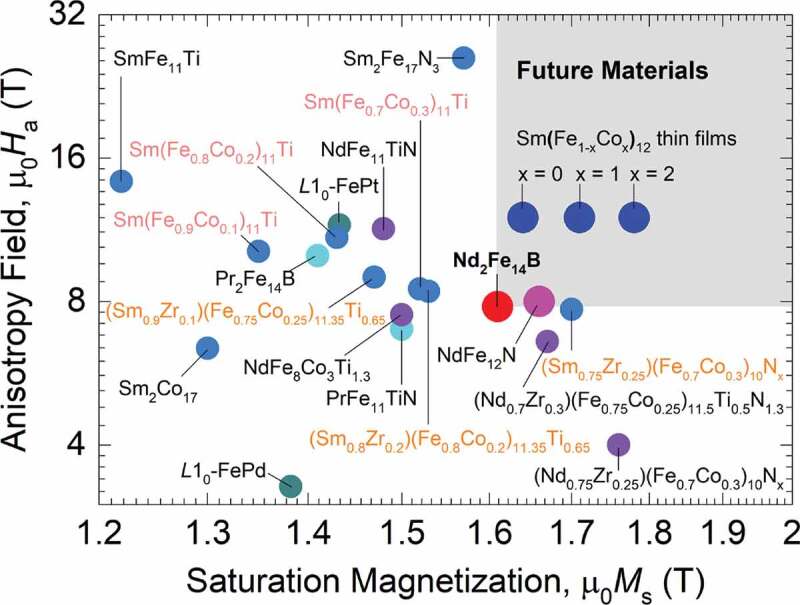
Room-temperature intrinsic magnetic properties of up-to-date representative compounds. The Sm(Fe_1-*x*_Co*_x_*)_12_ and NdFe_12_N compounds are in the form of thin film; the other compounds are in the form of micropowders. Data are incorporated with references [1,11–22]

The current high-end permanent magnet, Nd_2_Fe_14_B (*P*4_2_/*mnm*) compound, has a relatively low Curie temperature $T_{c}$ of 313°C [1,2] and, since its sintered magnet (Nd_2_Fe_14_B: 0.982 vol.%, O_2_: 600 ppm, grain orientation: 0.991) has reached the room-temperature ${\left(BH\right)}_{max}$ of 474 $kJm^{-3}$ [3], approaching the theoretical limit of 520 $kJm^{-3}$, high-performance permanent magnetic compounds that outperform the Nd_2_Fe_14_B and operate at elevated temperatures (typically 200°C) for highly efficient electric motors and generators are ever-increasing demand [4–8]. Uniaxial magnetocrystalline multielement R–T intermetallics are arguably the exclusive candidates that can process ultra-large intrinsic magnetic properties, where strong spin-orbit coupling (SOC) of 4*f* electrons of R sublattice originates large uniaxial magnetocrystalline anisotropy field, $H_{a}$, and large magnetic moment and strong exchange interactions of 3*d* electrons of T sublattice result in large saturation magnetization, $M_{s}$, and high $T_{c}$, respectively, [9,10]. Among them, R_2_T_17_ (Th_2_Zn_17_-type, *R*-3*m*) and RT_12_ (ThMn_12_-type, *I*4/*mmm*) compounds have the potential to meet current demands for rare-earth-element-lean PMs, owing to their intrinsic magnetic properties superior to those of the Nd_2_Fe_14_B compound (Figure 1) [1,2,11–22].

**Figure 2.**
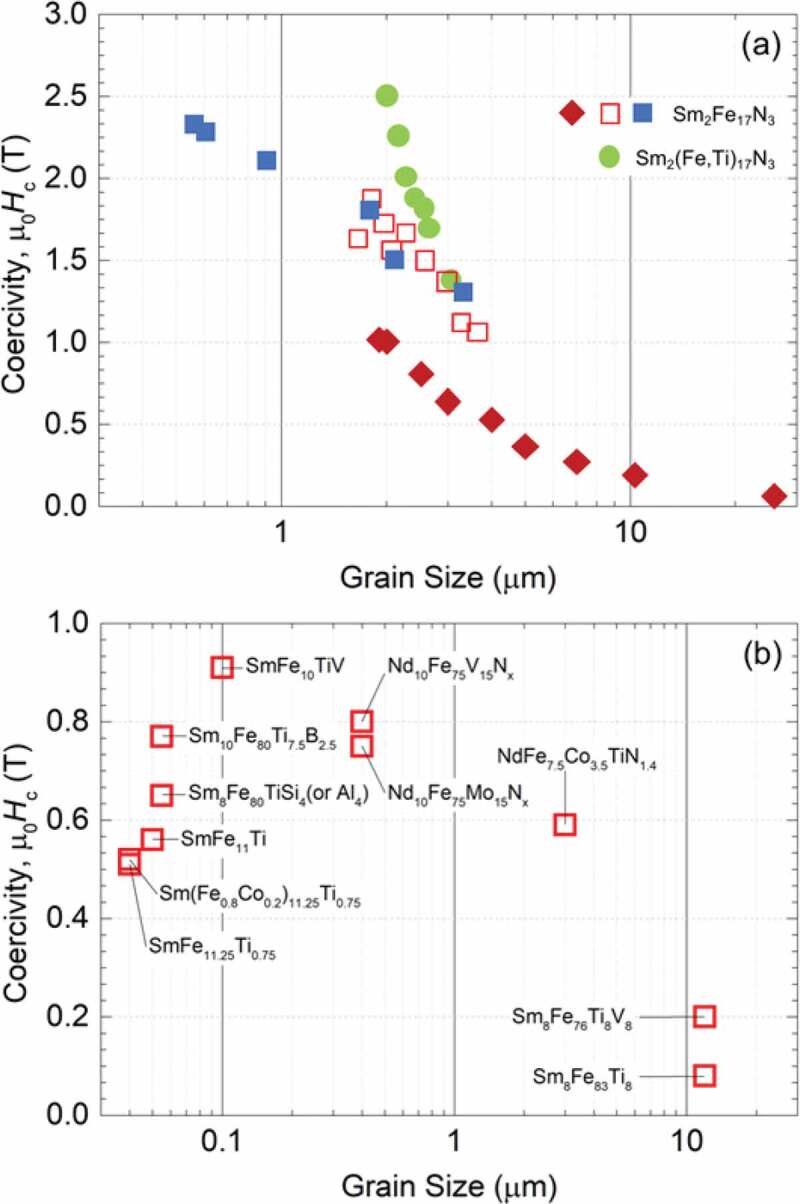
Grain size dependence of room-temperature coercivity. (a) Sm_2_Fe_17_N_3_ compounds. (b) R(Fe,M)_12_X_x_ (R = Nd, Sm; M = Ti, V, Co, Mo, Al, Si: phase stabilizing elements; X = N, B) compounds. (a) Reprinted with permission from [39]. Copyright 2020 Elsevier. (b) Data are incorporated with references [40–50]

Magnetism on the mesoscopic scale, which is known as micromagnetism, exhibits particularly rich extrinsic behavior. $H_{c}$ is an extrinsic property of crucial importance in permanent magnetism and is governed by the real structure of materials under Brown’s paradox [23,24]: $H_{c}$ is reduced to $\alpha H_{a}$ by defects, where the factor α (0 ≤ α < 1) describes microstructural details [25–29]. The MMPs, especially magnetic nanoparticles (MNPs), are an important class of magnet building blocks that can be used to fabricate high-performance anisotropic PMs [30–32]. Their unique feature is the size-dependent coercivity: $H_{c}$ of a single-domain grain increases beyond the superparamagnetic critical size ($D_{sp}$) as $H_{c}∼1-{\left(D_{sp}/D\right)}^{3/2}$, reaches the maximum at the single-domain critical size ($D_{sd}$) given by $D_{sd}\approx 72\sqrt{A_{ex}K_{1}}/\mu_{0}M_{s}^{2}$ where $A_{ex}$ is the exchange stiffness and $K_{1}$ is the first anisotropy constant, and then decreases as $H_{c}∼1/D^{n}$, provided that the grain has a strong cubic anisotropy [33–36]. The grain-size dependent coercivities of representative R_2_T_17_ and RT_12_ compounds are named a few and shown in Figure 2 [37–50]. Owing to the phase-stabilization challenges, control over the microstructure of R–T multielement materials is still a non-trivial task, though the R–T permanent magnetic materials have been established since 1960s [51] and the Nd_2_Fe_14_B compound has been utilized since its discovery in 1984 [52–55].

Synthesis of multielement R–T intermetallics, especially the RT_12_ compound, is very challenging due to their complex crystal structure, desired phases formed in narrow compositions and at very high temperatures (700–1200°C), and poor chemical stability in the air environment [56–60]. In general, the R–T intermetallics with equilibrium phases can be synthesized by equilibrium processes under the framework of their equilibrium phase diagrams, such as cooling of the alloying liquid with a very low cooling rate and annealing of the as-casting ingots at elevated temperatures for a time as long as possible. The later process utilizing arc-melting or induction-melting and subsequent annealing is convenient to synthesize the intermetallics [12,13,16–19,22,37,58,59,61]. However, the equilibrium processes often lead to the formation of impurities because the strict equilibrium conditions to give pure phases are hardly realized, and/or the metals R and T can easily form several equilibrium phases, thus make the microstructure, especially the size, less controllable. In contrast, the nonequilibrium processes are appropriate for synthesizing not only metastable compounds but also the intermetallics with desired crystal structures free from impurities and a fine grain microstructure [61]. The most typical method is first to form the amorphous phase, followed by annealing at an appropriate temperature [14,40–50]. The annealing evolves the formation of metastable phases, which can be produced at various extreme nonequilibrium conditions, and the dynamical transformation between the metastable and the equilibrium phases, corresponding to the local free-energy minima. The differences between the crystallographic symmetries of the phases result in the differences in the local free-energy minima for the formation of the phases. Other factors, such as the composition and the atomic binding energy, of course, also play an important role in the formation and stability of the phases. The descending sequence of the symmetries for the structures of the R–T compounds has been found as follows: Amorphous, CaCu_5_-type (*P*6/*mmm*), TbCu_7_-type (*P*6/*mmm*), Th_2_Ni_17_-type (*P*6_3_/*mmc*), Nd_2_Fe_14_B-type (*P*4_2_/*mnm*), ThMn_12_-type (*I*4/*mmm*), Th_2_Zn_17_-type (*R*-3*m*), and Nd_3_(Fe,Ti)_29_-type (*A*_2_/*m*) [56,57,61]. The differences between the free-energy minima for the formation of the last five equilibrium phases in the sequence may be quite small, depending on the composition of alloys and the condition of the synthetic process, and, thus only one equilibrium phase usually forms as the final one under a given condition of compositions and processes [61]. The CaCu_5_-type (*P*6/*mmm*) structure with the highest symmetry among those of the R–T metastable and equilibrium phases is the basic one from which the structures of various R–T compounds can be derived by replacements of the R atoms with a pair of T atoms, which is known as dumbbell atoms, as follows [56,57]:

$$\begin{array}{c} 2RT_{5}-R+2T=RT_{12}\left(I4/mmm\right),\\ 3RT_{5}-R+2T=R_{2}T_{17}\left(R\text{-}3m\;or\;P6_{3}/mmc\right),\\ 5RT_{5}-2R+4T=R_{3}T_{29}\left(A_{2}/m\right). \end{array}$$

For most of the R–T compounds, the metastable CaCu_5_-type phases may form in a narrow temperature range and, thus, are hardly observed experimentally. The formation of the CaCu_5_-type phases can be observed by careful annealing with a very slow heating rate for a short time [46], whereas the metastable TbCu_7_-type phases form in a sufficiently wide temperature range to be observed in various synthetic processes [14,40,43]. In practice, the metastable compounds usually crystallize at annealing temperatures slightly higher than the crystallization temperature of the amorphous phase, and subsequently can be dynamically transformed into more stable compounds at higher annealing temperatures [61]. Therefore, the choice of the appropriate annealing temperatures under a given condition of compositions and processes is essential to promote the formation of the desired intermetallic compounds. The following processes are those based on the method above: mechanical alloying; mechanical milling including high-energy ball milling (HEBM) and surfactant-assisted ball milling (SABM); rapid quenching/melt-spinning; hydrogenation disproportionation desorption recombination (HDDR) [61]. They are popularly adopted for the massive production of fine powders, although they have limited control of the microstructure of materials. DC magnetron sputtering nonequilibrium process is often used to fabricate metastable RT_12_ films [15,20]. Solid-state and solid-gas reactions are the other two powerful nonequilibrium processes for the synthesis of the metastable and intermetallic compounds [19,37–39], and they are further discussed hereinafter. Among bottom-up synthetic approaches, chemical synthesis is the most versatile method for the preparation of MMPs with controllable composition, structure, size, and shape [30–32,62]. Recent advances in the solution-phase synthesis of MNPs followed by the solid-state reaction have overcome the phase stabilization challenges in preparing R–T intermetallics, leading to the successful synthesis of some binary R–T MMPs (e.g., SmCo_5_, Sm_2_Co_17_) with ultra-large room-temperature $H_{c}$ [62,63]. Herein we present an overview of our ongoing research in the context of other recent developments in the chemical synthesis of the most challenging multielement pseudobinary R_2_T_17_ and RT_12_ intermetallics with an emphasis on grain size and composition control. This review covers the basis behind the use of nanoparticles (NPs) as precursors for the microstructure control of MMPs and presents the most recent results of Sm_2_Fe_17_N_3_ and (R,Zr)(Fe,Co,M)_12_ (R = Nd, Sm; M = Ti, V, Cr, Mn, Co, Mo, W, Al, Si, Ga) MMPs. The review also summarizes the efforts in the chemical synthesis of magnetically hard/soft exchange-coupled R–T/T NCMs.

## 2. Synthesis of nanostructured precursors

The high negative reduction potentials of R cations, a large difference in reduction potentials of R and T cations, and high chemical instability of R metals make it impossible to directly synthesize R–T intermetallics by solution-phase chemical reactions. An alternative chemical synthetic approach is to first synthesize nanostructured precursors, which are chemically stable and readily synthesized by solution-phase reactions, followed by R–D reactions of the precursors. Monodisperse nanostructured precursors with controllable composition, size, and shape are an important key to determine the microstructure of MMPs, and they are advantageous depending on their structural fashions such as core@shell, encapsulated, doped, or mixed oxide NPs.

**Figure 3.**
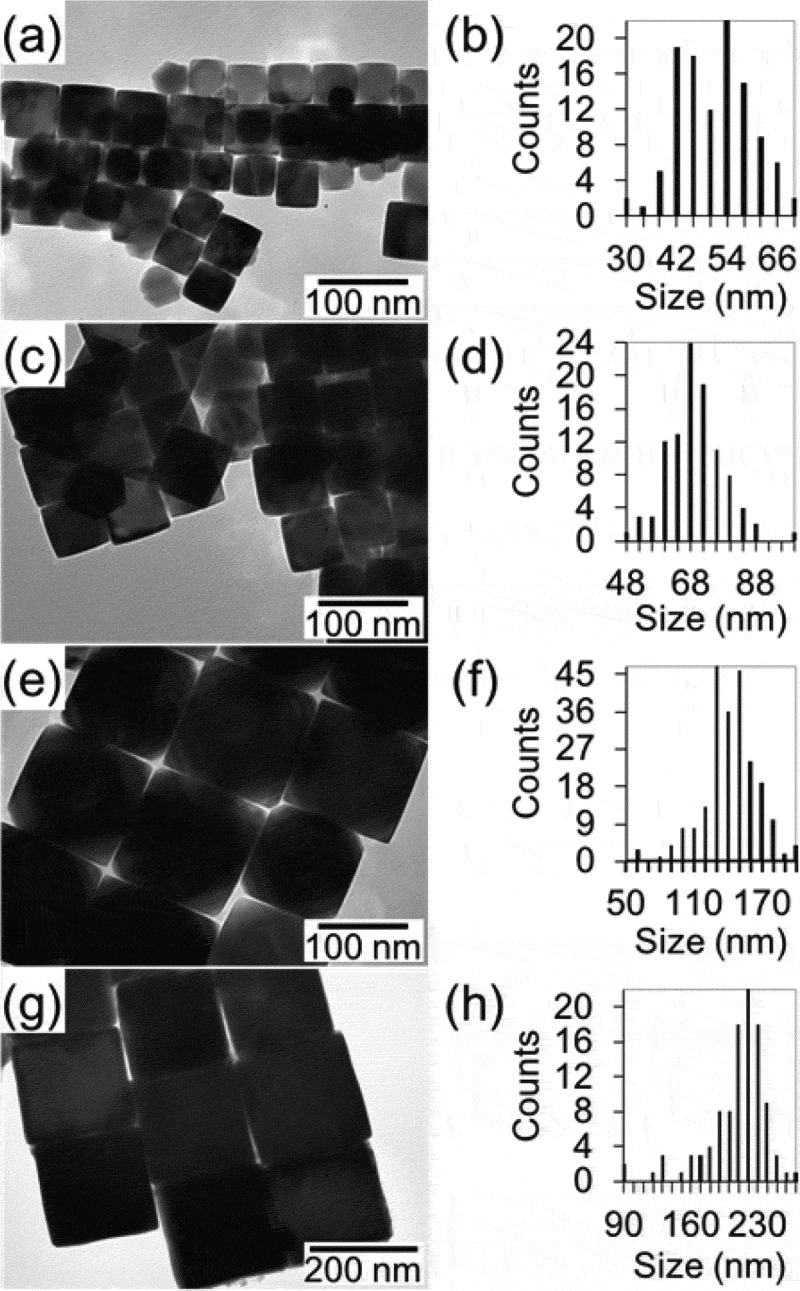
Size evolution of Fe_3_O_4_ (*Fd*-3*m*) NPs. (a,c,e,g) Transmission electron microscopy (TEM) images and (b,d,f,h) size distributions. Reprinted with permission from [70]. Copyright 2020 Wiley VCH

**Figure 4.**
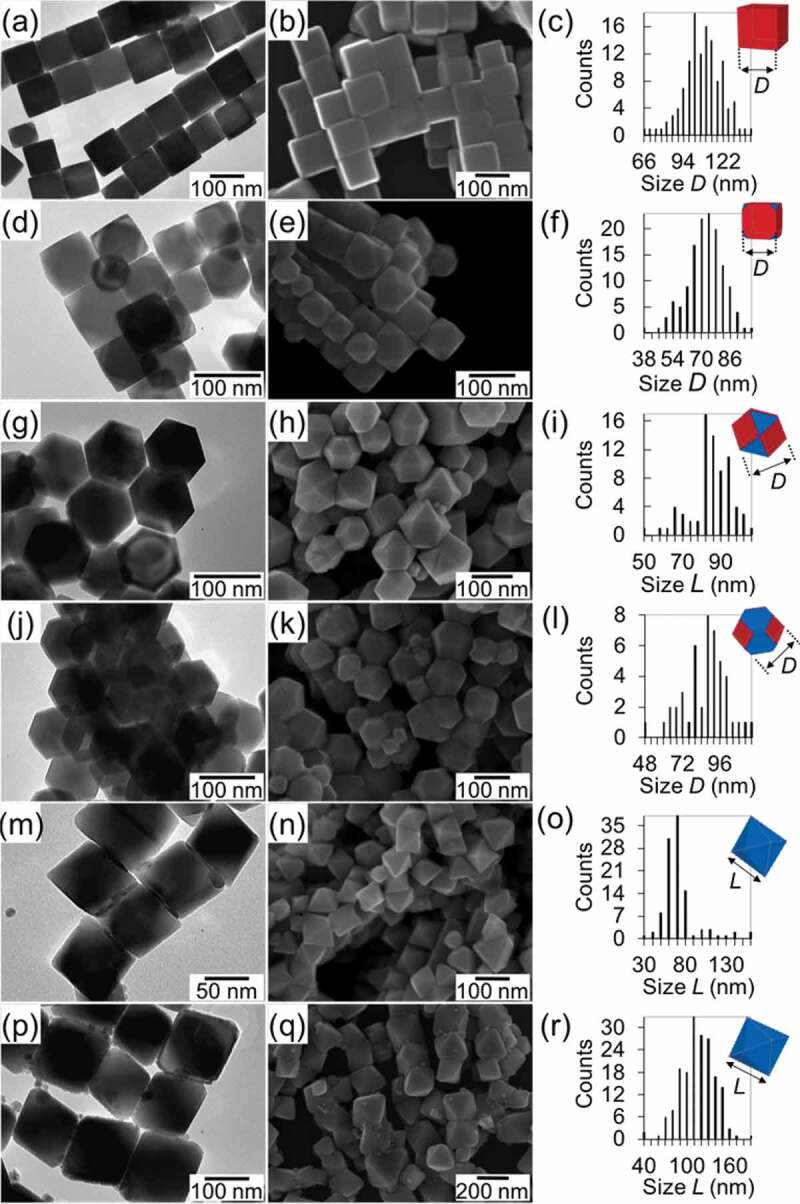
Shape evolution of Fe_3_O_4_ (*Fd*-3*m*) NPs: (a–c) cubes, (d–f) truncated cubes, (g–i) cuboctahedra, (j–l) truncated octahedra, (m–o) octahedra with small size, and (p–r) octahedra with large size. (a,d,g,j,m,p) TEM images, (b,e,h,k,n,q) Scanning electron microscopy (SEM) images, and (c,f,i,l,o,r) size distributions. Insets show the {100} and {111} planes in red and blue, respectively. Reprinted with permission from [70]. Copyright 2020 Wiley VCH

**Figure 5.**
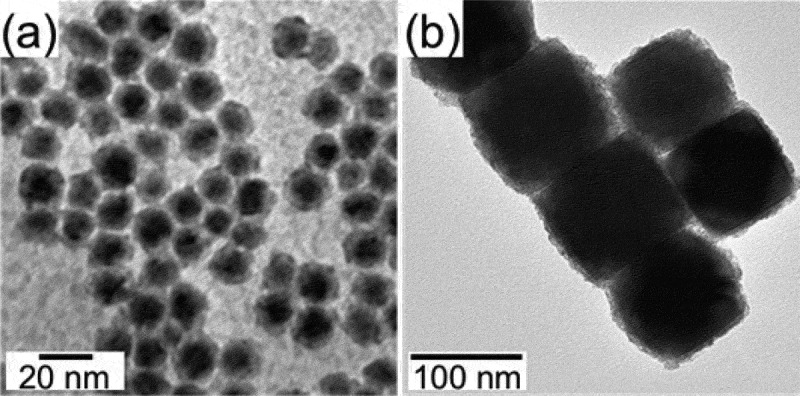
Chemically-synthesized core@shell nanostructured precursors. (a,b) TEM images of Co@Sm–O (Co core: *Fm*-3*m*, 8 nm; Sm:Co = 1:4.3 at%) (a) and Fe_3_O_4_@Sm–O (Fe_3_O_4_ core: *Fd*-3*m*, 79 nm; Sm:Fe = 1:11.5 at%) (b). (a) Reprinted with permission from [65]. Copyright 2020 Wiley VCH. (b) Reproduced with permission from [72]. Copyright 2020 the Chemical Society of Japan

The core@shell nanostructures composed of T metal or T oxide (T–O) cores and R oxide (R–O) shells, namely T/T–O@R–O NPs hereinafter, are usually synthesized *via* a two-step reaction: the T or T–O NPs are firstly synthesized, followed by the deposition of R–O over the surface of the T or T–O NPs. The Co (*Fm*-3*m*) and amorphous Fe NPs with particle sizes of < 10 nm were readily synthesized by thermal decomposition of Co_2_(CO)_8_ and Fe(CO)_5_, respectively, as reported by Sun and co-workers [64,65]. The Co (*P6*_3_/*mmc*) nanorods with the length in the range of 200–300 nm and the average diameter of 20 nm could be synthesized by using a Ru-catalyzed solvothermal reaction of cobalt laurate in the presence of hexadexylamine in 1,2-butanediol [66]. Amorphous Fe nanospheres with a particle size of 200 nm were synthesized by Carpenter and co-workers [67], where FeSO_4_ was reduced to Fe by NaBH_4_ at room temperature in the presence of sodium citrate. A series of Fe–O NPs with tunable sizes in a wide range from *ca*. 10 nm to several hundred nanometers were also successfully synthesized by the following procedures. Wüstite FeO (*Fm*-3*m*) NPs were synthesized by reductive thermal decomposition of Fe(acac)_3_ (acac = acetylacetonate) with oleic acid (OA) and oleylamine (OAm); their sizes were tuned from 14 to 100 nm by controlling the reaction temperatures [68]. Magnetite Fe_3_O_4_ (*Fd*-3*m*) nanocubes were synthesized by thermal decomposition of Fe(acac)_3_ in the presence of OA in benzyl ether solvent at 290°C [69]; their sizes were tuned from 20 to 200 nm by varying the OA concentration, as shown in Figure 3 [70]. Hyeon and co-workers developed the ultra-large-scale synthesis of smaller Fe_3_O_4_ nanospheres with tunable sizes in the range of 5–20 nm by thermal decomposition of Fe(III) oleate in various solvents with different boiling points [71]. The reactivity of nanostructured precursors toward the following R–D reaction can be tailored by adopting NPs with different shapes as the surface energy can govern the inter-diffusion of metal atoms. For this purpose, Fe_3_O_4_ (*Fd*-3*m*) NPs with rationally-controlled shapes from a cube, truncated cube, cuboctahedron, truncated octahedron, to octahedron were prepared by varying the concentration of CPC (CPC = cetylpyridinium chloride), as shown in Figure 4 [70]. Once the transition metal core like the Co core was prepared, amorphous Sm–O shell could be deposited by thermal decomposition of Sm(acac)_3_ at 300°C in 1-octadecene solvent, as shown in Figure 5(a) [65,66]. The temperature rate was kept as low as 2°C min^–1^ to avoid homogeneous nucleation of particulate Sm–O NPs. The method has been successfully extended to synthesize Fe_3_O_4_@Sm–O NPs, as shown in Figure 5(b) [72]. The molar ratio of Fe to Sm could be tuned by adjusting relative amounts of Sm(acac)_3_ and Co or Fe_3_O_4_ NPs.

**Figure 6.**
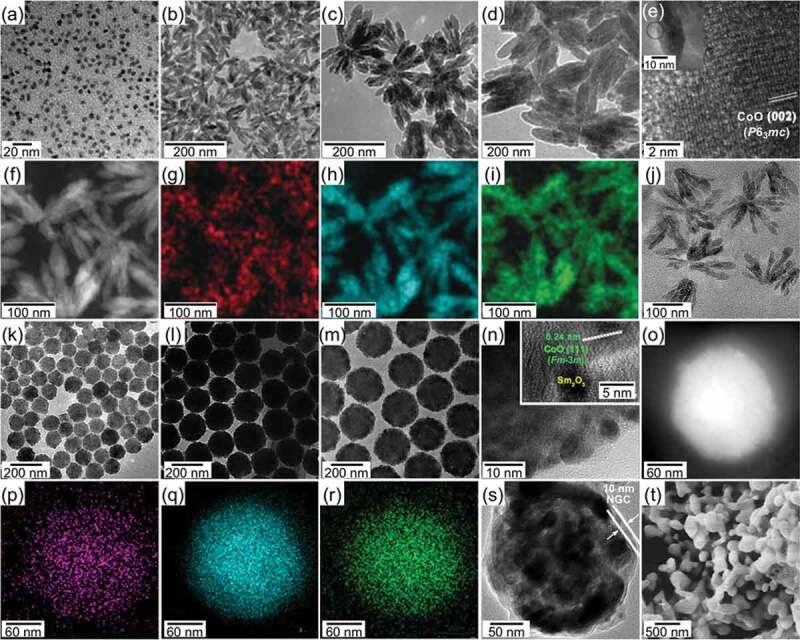
Chemically-synthesized nanostructured precursors. (a) TEM image of Sm–O encapsulated CoO (*Fm*-3*m*) NPs (7 nm, Sm:Co = 1:3.6). (b–d) TEM images of Sm–O encapsulated CoO (*P*6_3_*mc*) multipods (Sm:Co = 1:4.5): (b) 60 ±10 ×10 ±3 nm, (c) 110 ±20 ×25 ±5 nm, and 220 ±40 ×45 ±5 nm. (e) HRTEM (high-resolution TEM) image of an enlarged part of a nanorod (inserted). (f) HAADF-STEM (high-angle annular dark-field scanning TEM) image and (g–i) elemental mapping images of Sm (g), Co (h), and O (i) of the multipods shown in (c). (j) TEM image of the multipods (shown in (c)) imbedded into a CaO matrix. (k–m) TEM images of Sm–O encapsulated CoO (*Fm*-3 *m*) NPs: (k) 110 ±8 nm, (l) 150 ±12 nm, (m) and 200 ±15 nm. (n) HRTEM image of a section of one 200 nm NP shown in (m), showing a mixture of smaller CoO and Sm–O NPs. (o) HAADF-STEM image and (p–r) elemental mapping images of Sm (p), Co (q), and O (r) of one representative 200 nm NP shown in (m). (s) TEM image of one 200 nm NP (shown in (m)) coated with a 10 nm layer of NGC (N-doped graphitic carbon). (t) SEM image of Fe_2_O_3_, NdFeO_3_, and Fe_2_(MoO_4_)_3_ mixed NPs (*ca*. 200 nm). (a) Reproduced with permission from [73]. Copyright 2020 The Royal Society of Chemistry. (b–j) Reprinted with permission from [74]. Copyright 2020 Wiley VCH. (k–t) Reproduced with permission from [75,98]. Copyright 2020 American Chemical Society

The T/T–O@R–O nanostructures are highly desirable because their size can be fully tuned in the mesoscopic scale from a few nanometers to one micrometer by well-established solution chemical synthetic methods. However, this strategy has succeeded rather in binary R–T MMPs [65,66]; it may be inappropriate for higher-multielement R–T MMPs since the synthetic process involves multi-step reactions giving a very low overall yield and the R/T ratio is not well-controlled. For better control of the R–T composition and full access to the control of particle size, the encapsulated nanostructures, where R–O and T–O in crystalline and/or amorphous forms are co-precipitated within one nanostructure (namely RT–O NPs hereinafter), can be used as precursors. The SmCo-O (7 nm) NPs were synthesized by thermal decomposition of Sm(OAc)_3_ and Co(OAc)_2_ (OAc = acetate) by Sun and co-workers, as shown in Figure 6(a) [73]. We successfully extended this method to synthesize multielement NPs, *e.g*., Sm–O, Zr–O, and Ti–O encapsulated CoFe_2_O_4_ (*Fd*-3*m*) NPs (5 nm), which will be reported in the forthcoming paper. Besides these, the Sm(Co or Fe)–O NPs with tunable sizes in the range of 60–220 nm and different morphologies were synthesized directly from thermal decomposition of Sm(acac)_3_ and Co/Fe(acac)_2_ (Figure 6(b–j)) [74] or SmCo–oleate complex (Figure 6(k–s)) [75]. The SmCo–O NPs with various morphologies, such as Sm(OH)_3_–Co nanorods [76] or urchin-like [77], Sm(OH)_3_–Co(OH)_2_ nanoflakes [78,79], and SmCo–O nanofibers [80,81], could be successfully obtained by sonification [76], hydro/solvothermal reaction [77–79], and electrospinning [80,81]. Once the R amounts in these encapsulated nanostructures are under the critical concentration for the solid-solution formation, the R-doped T–O nanostructures can be synthesized by using procedures which are similar to the above [81–85]. A series of R-doped Fe_2_O_3_ or Fe_3_O_4_ (R = Sm, Eu, Gd, Tb, Ho, Er, Y) NPs with different shapes and particle sizes tuned in the range of 5 nm–1 μm could be obtained by thermal decomposition [82,84], hydrothermal reaction [85], and ultrasonication [86]. These precursors with R compositions varied below 20 at.% are suitable for the synthesis of RT_12_ MMPs.

The most simple and robust strategy for the control over the composition of the multielement R–T MMPs is to prepare mixed NPs of R–O and T–O (namely [T–O,R–O] NPs hereinafter): the precursors are synthesized *via* one-pot solution chemical synthesis; their stoichiometry is well determined by the feeding ratio of starting materials. Mixtures of Sm_2_O_3_/Sm(OH)_3_, Co/Co–O/Co(OH)_2_/CoOOH, and/or Fe/Fe–O NPs were usually prepared by reductive thermal decomposition [87], solvothermal reaction [88], co-precipitation [89–96], and ultrasonication [97]. The sol-gel method is rather simple and efficiently produces a mixture of superfine oxide NPs, where a mixture of R–O and T–O gel is formed by using a poly-network gel process at elevated temperatures, followed by calcination to convert the gel to the superfine oxides [98–100]. By varying the concentration of the R and T ions in the gel, the size of oxide NPs could be controlled in a wide range from *ca*. 10 nm to several hundred nanometers, as shown in Figure 6(t) [98]. Monodisperse and size-tunable T/T–O@R–O and RT–O nanostructured precursors with homogeneous composition have opened horizons for optimization of the microstructure of R–T MMPs since they could be embedded in dispersant matrixes (e.g., CaO, graphite oxide GO), as shown in Figure 6(j) [74,78,79], or tightly coated with other layers intact (e.g., CaO, GO, nitrogen-doped graphitic carbon NGC) in core@shell structures, as shown in Figure 6(s) [73,75,76]. While mixed NPs strategy partially succeeded in only the embedding, leading to the limited kinetic control of the microstructure of R–T MMPs [88–92,100]. Although the mixed NPs exhibited poor control of particle size, they were capable to synthesize multielement R–T MMPs owing to feasible control of composition [98].

## 3. Synthesis of multielement R–T MMPs

Multielement R–T intermetallics, represented by Sm_2_Fe_17_N_3_, (Nd,Zr)(Fe,Co,Ti)_12_N, and (Sm,Zr)(Fe,Co,Ti)_12,_ with large $H_{a}$, large $M_{s}$, and high $T_{c}$ are the most promising candidates to replace Nd_2_Fe_14_B for modern permanent magnet applications (Figure 1). However, their chemical synthesis remains a great challenge due to the high negative reduction potentials of R cations (e.g., Sm^3+^: – 2.304 V, Nd^3+^: – 2.323 V) and very low chemical stability of R metals. Generally, the R and T cations are reduced by strong reducing agents (e.g., CaH_2_, Ca) accompanied by diffusion of R and T atoms to form R–T intermetallics under high-temperature solid-state reaction conditions, which is known as R–D process. Before the R–D process, it may need to remove organics from nanostructured precursors by calcination to avoid any undesirable formation of carbides and/or C interstitial compounds in the following R–D reaction, and/or adopt H_2_ pre-reduction of the calcined precursors to promote the R–D reaction. In this synthesis, the R–T MMPs encounter common issues of sintering in the R–D reactions at high temperatures and oxidation in air environments. A great strategy is the coating of nanostructured precursors with stable materials, which were mentioned in Section 2, to stabilize MMPs formed in high-temperature solid-state reaction conditions and against oxidation in air. The chemical synthetic approach has been successful to prepare rather simple binary R–T MMPs. Indeed, the SmCo_5_ and Sm_2_Co_17_ MMPs with tunable sizes in a wide range from a few nanometers to a few micrometers have been successfully synthesized [65,66,73–78,80,81,87–93,97,99,100]. The resultant MMPs were dispersible in common solvents [74,75,91,100], possessed ultra-large $H_{c}$ reaching the highest yet reported room-temperature $\mu_{0}H_{c}$ of 7.2 T [91] for any permanent magnetic materials, and were strongly stable against oxidation at elevated temperatures [75]. The chemical synthesis of SmCo_5_ MMPs has been well documented in a previous review [63]. Thus, the nanostructured precursors are readily prepared by a wide range of solution chemical synthetic methods and the intriguing results of chemically-synthesized SmCo_5_ MMPs are triggering the chemical synthesis of MMPs of Sm_2_Fe_17_N and (R,Zr)(Fe,Co,M)_12_ (R = Nd, Sm; M = Ti, V, Cr, Mn, Co, Mo, W, Al, Si, Ga) compounds. The chemically synthesized MMPs of these compounds and their room-temperature magnetic properties are summarized in Table 1.

**Table 1.** Synthetic details and room-temperature magnetic properties of Sm_2_Fe_17_N and (R,Zr)(Fe,Co,M)_12_ (R = Nd, Sm; M = Ti, V, Cr, Mn, Co, Mo, W, Al, Si, Ga) MMPs synthesized by a reduction–diffusion (R–D) process. ^①^T/T–O@R–O NPs: core@shell structure, RT–O NPs: encapsulation, and [T–O,R–O] NPs: mixture of oxide NPs. ^②^Gas-solid nitridation. *High pressure. ^③^Solid-state nitridation. ^④^Dehydrogenation after the rinse with H_2_O. ^⑤^Slow-oxidation of Ca residue before the rinse with H_2_O. A typical chemically-synthetic procedure for the synthesis of multielement R–T MMPs composed of calcination, pre-reduction by H_2_, and (R–D) process using Ca or CaH_2_ as reductant

Materials	Nanostructured precursors		Calcination temperature (°C)	H_2_ reduction temperature (°C)	R–D reaction(Nitridation)temperature(°C)	MMPs Size(μm)	$T_{c}$(°C)	$M_{s}$(emu g^−1^)	$\mu_{0}H_{a}$(T)	$M_{r}$(emu g^−1^)	$\mu_{0}H_{c}$(T)	Ref.
	Structural fashion^①^	Size(nm)										
R_2_Fe_17_ (Th_2_Zn_17_, *R*-3 *m*)												
Sm_2_Fe_17_N	[Fe_2_O_3_,Fe_3_O_4_,SmFeO_3_]	*ca*. 100	500	700	900(430^②^)	0.7 ±0.0	–	–	–	–	2.32	[39]
		–	800	900	900(430^②^)	1.9 ±0.8	–	–	–	–	*ca*. 1.8	
		–	1000	900	1000(430^②^)	3.5 ±1.3	–	–	–	–	*ca*. 1.3	
	Fe_3_O_4_@Sm–O NPs	*ca*. 30	1000	900	900(435^③^)	1.9 ±1.0	–	–	–	–	1.3	[72]
	SmFe–O NPs	110 ±20	185	N/A	850(600^②^)	0.10 ±0.02	–	127.9	–	–	1.54	[74]
	[Fe–O,Sm–O] NPs	–	N/A	800	900(420^②^)	0.47 ±0.09	–	132	–	89	2.47^④^	[95]
				700	900(420^②^)	0.6 ±0.2	–	134	–	100	2.28^④^	
					950(420^②^)	0.9 ±0.3	–	142	–	110	1.81^④^	
				600	900(420^②^)	0.6	–	–	–	–	2.78^⑤^	[96]
					950(420^②^)	0.9	–	–	–	–	2.37^⑤^	
					980(420^②^)	1.5	–	–	–	–	2.01^⑤^	
RFe_12_ (ThMn_12_, *I*4/*mmm*)												
NdFe_10_Mo_2_	[Fe_2_O_3_,NdFeO_3_,Fe_2_(MoO_4_)_3_]	*ca*. 200	500	700	1010	3–8	–	–	–	–	–	[98]
NdFe_10_Mo_2_N	[Fe_2_O_3_,NdFeO_3_,Fe_2_(MoO_4_)_3_]	*ca*. 200	500	700	1010 (550–600^②*^)	3–8	360	–	–	–	0.35	[131]
NdFe_10_Mo_2_	[Fe_2_O_3_,Nd_2_O_3_,MoO_3_,TiO_2_]	–	500	700–800	1100 (550–600^②*^)	–	453	65	–	–	–	[135,136]
NdFe_10_Mo_2_H*_x_*							478	91	–	–	–	
NdFe_10_Mo_2_N*_x_*							553	92	7.2	–	–	
NdFe_10.25_Mo_1.5_Ti_0.25_							483	89	0.04	–	–	
NdFe_10.25_Mo_1.5_Ti_0.25_H*_x_*							488	93	0.04	–	–	
NdFe_10.25_Mo_1.5_Ti_0.25_N*_x_*							573	103	8.2	–	–	
NdFe_10.5_MoTi_0.5_							498	109	1.1	–	–	
NdFe_10.5_MoTi_0.5_H*_x_*							508	114	1.1	–	–	
NdFe_10.5_MoTi_0.5_N*_x_*							608	118	–	–	–	
NdFe_10.75_Mo_0.5_Ti_0.75_							523	122	1.4	–	–	
NdFe_10.75_Mo_0.5_Ti_0.75_H*_x_*							533	125	1.4	–	–	
NdFe_10.75_Mo_0.5_Ti_0.75_N*_x_*							673	130	–	–	–	
NdFe_11_Ti							533	124	1.6	–	–	
NdFe_11_TiH*_x_*							563	129	2.2	–	–	
NdFe_11_TiN*_x_*							693	127	8.0	–	–	

### 3.1. Sm_2_Fe_17_N MMPs

**Figure 7.**
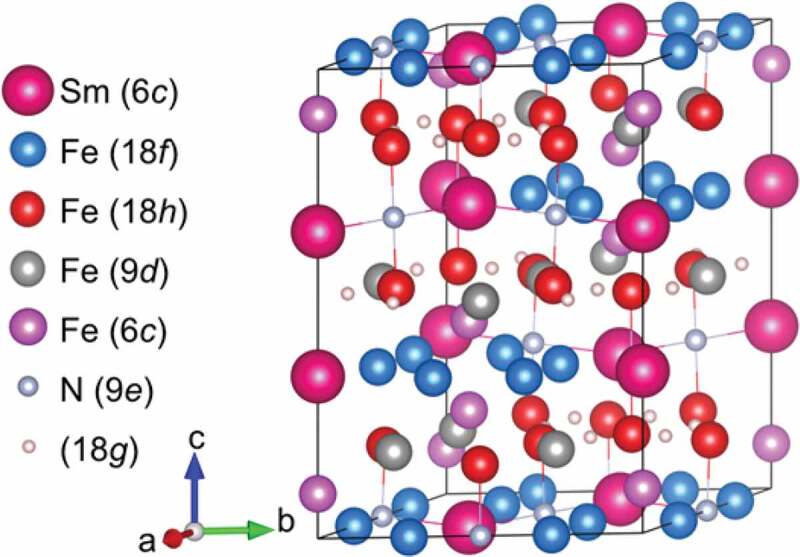
Crystal structure of Sm_2_Fe_17_N_3_ (Th_2_Zn_17_, *R*-3*m*) compound

**Figure 8.**
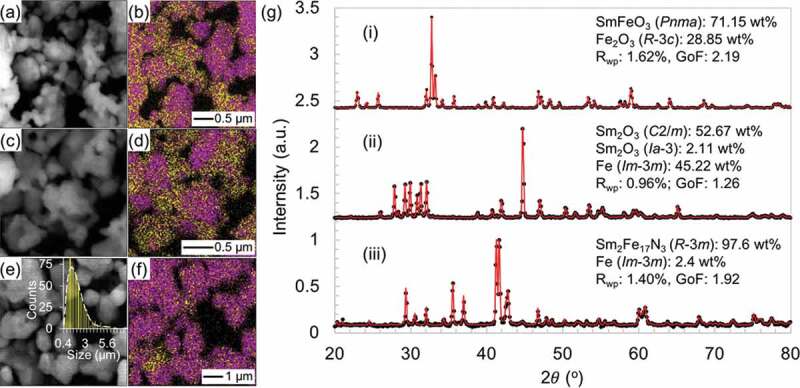
Structural analysis of chemically synthesized Sm_2_Fe_17_N_3_ MMPs (*R*-3*m*, 1.9 ±1.0 μm). (a,c,e) SEM images, (b,d,f) energy-dispersive spectroscopy elemental maps (Fe K edge: purple, Sm L edge: yellow), and (g) Rietveld refinement XRD patterns of Fe_3_O_4_@Sm–O NPs calcined at 1000°C for 1 h (a,b,g(i)), subsequently reduced by H_2_ at 900°C for 1 h (c,d,g(ii)), and Sm_2_Fe_17_N_3_ MMPs synthesized by Ca R–D at 900°C for 1 h in Ar and *in situ* nitridation at 435 °C for 10 h in N_2_ (e,f,g(iii)). Inset in (e) shows the grain size distribution of the Sm_2_Fe_17_N_3_ MMPs. Reproduced with permission from [72]. Copyright 2020 the Chemical Society of Japan

The Sm_2_Fe_17_ (Th_2_Zn_17_-type, *R*-3 *m*) compound, representative to the R_2_T_17_ intermetallic series, has a relatively low $\mu_{0}M_{s}$ = 1.03 T, very low $\mu_{0}H_{a}$ < 1 T, and very low $T_{c}$ = 116°C [100–102]. The interstitial doping of the Sm_2_Fe_17_ compound with nitrogen atoms forms the Sm_2_Fe_17_N_3_ (Th_2_Zn_17_-type, *R*-3 *m*) compound [19,101–103]; its intrinsic magnetic properties enormously increases to $\mu_{0}M_{s}$ = 1.57 T, $\mu_{0}H_{a}$ = 26 T, and $T_{c}$ = 473°C [19], superior to those of the Nd_2_Fe_14_B compound (Figure 1). Nitrogen atoms interstitially doped in the 9*e* octahedral sites (Figure 7) expand the unit-cell volume and increase the Fe–Fe exchange interactions, resulting in the increases in $M_{s}$ and $T_{c}$, respectively [102–104]. The hybridization of Sm-*f* states and N-*p* states changes magnetization direction from the easy-plane in the Sm_2_Fe_17_ to the easy-*c* axis in the Sm_2_Fe_17_N_3_ and affects band energy, leading to a large uniaxial magnetocrystalline anisotropy [104]. The Sm_2_Fe_17_N_3_ phase is metastable and, thus synthesized by nitridation of the Sm_2_Fe_17_ with N_2_ or NH_3_ in gas-solid reaction or with melamine (C_3_H_6_N_6_) in a solid-state reaction under high temperature and/or pressure conditions. In practice [72], the Fe_3_O_4_@Sm–O NPs (*ca*. 30 nm) described in Section 2 (Figure 5(b)) were calcined at 1000°C for 1 h in air (Figure 8(a,b,g(i))), and subsequently reduced by H_2_ at 900°C for 1 h (Figure 8(c,d,g(ii))). The resultant NPs were converted into Sm_2_Fe_17_ MMPs by the Ca R–D reaction at 900°C for 1 h in Ar, subsequently converted into Sm_2_Fe_17_N_3_ MMPs by *in situ* nitridation at various temperatures for 10 h in N_2_ (Figure 8(e,f,g(iii))). The $H_{c}$ of Sm_2_Fe_17_N_3_ MMPs depended on the nitriding temperature, as shown in Figure 8. This is related to thermal transformations in the nitridation process: nitridation in a gas-solid reaction can only proceed at relatively high temperatures (400–500°C); nitrogen concentration in Sm_2_Fe_17_N*_x_* (0 < *x* ≤ 3) is a function of nitriding temperature at low temperatures, while the Sm_2_Fe_17_N_3_ decomposed into SmN*_x_* and Fe at high temperatures [19,105,106]. For example, the resultant Sm_2_Fe_17_N_3_ MMPs formed by the nitridation at 435°C have an average particle size of 1.9 ±1.0 μm and a room-temperature $\mu_{0}H_{c}$ of 1.3 T in an isotropic sample before the rinse with H_2_O, as shown in Figures 8(e,f,g(iii)) and 9.

In the light of nanostructured precursors, the particle size of Sm_2_Fe_17_N_3_ MMPs can be controlled through thermodynamics based on careful observation of reaction temperature [39]. To this end, a synthesis using a mixture of Fe–O and Sm–O NPs prepared by a sol-gel method, as described in Section 2, was conducted *via* consecutive calcination, H_2_ pre-reduction, and Ca R–D at various temperatures in the ranges of 500–1000°C, 700–900°C, and 900–1000°C, respectively, followed by *in situ* nitridation at 430 °C. The particle size of the resultant Sm_2_Fe_17_N_3_ MMPs was largely dependent on the processing temperature. The first two processes conducted at low temperatures were crucially important to control the final MMP size at the following R–D process as their resultant small NPs facilitated the Ca R–D reaction at low temperatures to obtain small MMPs. The Ca R–D process, in its turn, effectively determined the particle size of the resultant Sm_2_Fe_17_N_3_ MMPs as higher R–D reaction temperatures led to larger MMPs due to the sintering of the particles. As a result, the Sm_2_Fe_17_N_3_ MMPs exhibited particle sizes tuned in the range of 0.7–3.5 μm and room-temperature $\mu_{0}H_{c}$ tuned in the range of 1.3–2.32 T in anisotropic samples. The smallest size of 0.69 μm was obtained at the lowest temperatures for the calcination at 500°C, H_2_ pre-reduction at 700°C, and Ca R–D processes at 900°C. The $H_{c}$ showed an obvious manifestation of size dependence and its highest $\mu_{0}H_{c}$ of 2.32 T was obtained for the smallest size of 0.69 μm (Figure 2(a), the blue rectangles). However, the syntheses above required an excessive amount of Sm by 25–30 at.% to the 2:17 stoichiometry to compensate for Sm evaporation during the Ca R–D reaction. The thermodynamic control could not proceed to obtain much smaller sizes as the calciothermic reduction must be conducted at temperatures far above the melting point of Ca (845°C). Recently, an efficient kinetic approach that was advanced by the engineering of nanostructured precursors could achieve the formation of Sm_2_Fe_17_N_3_ MMPs with better-controlled size and composition by Sun and co-workers [74]. In this synthesis, SmFe–O nanocubes (110 nm) were tightly coated with CaO by thermal decomposition of Ca(acac)_2_ at 200°C, calcined at 185°C for 5 h, and reduced by Ca at 850°C for 30 min in Ar. The resultant Sm_2_Fe_17_ MMPs were then mixed with C_3_H_6_N_6_ and annealed at 600°C for 6 h in Ar to form 100 nm Sm_2_Fe_17_N_3_ MMPs. The overall synthesis costed an excessive Sm composition of the SmFe–O nanocubes by only 2 at.% to the 2:17 stoichiometry to compensate for Sm loss. The resultant Sm_2_Fe_17_N_3_ MMPs were well dispersible in conventional solvents and exhibited room-temperature $\mu_{0}H_{c}$ of 1.54 T in an anisotropic PEG–embedded Sm_2_Fe_17_N_3_ (PEG = polyethylene glycol) sample. This $H_{c}$ value was smaller than that reported above because their particle size (100 nm) was smaller than the single-domain critical diameter of Sm_2_Fe_17_N_3_, *D*_sd_ = *ca*. 390 nm [36]. This method would give the Sm_2_Fe_17_N_3_ MMPs with larger sizes approaching the *D*_sd_ by increasing the particle size of the precursors, leading to ultra-large $H_{c}$.

**Figure 9.**
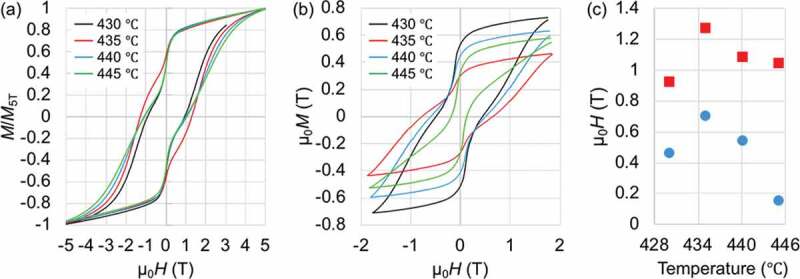
Room-temperature magnetic properties of Sm_2_Fe_17_N_3_ MMPs (*R*-3 *m*, 1.9 ±1.0 μm) formed by *in situ* nitridation of Sm_2_Fe_17_ MMPs at various temperatures for 10 h. (a,b) M-H curves before (a) and after (b) the rinse with H_2_O. (c) Nitriding-temperature dependence of coercivity (red rectangles: before the rise with H_2_O, blue circles: after the rinse with H_2_O). Replotted with permission from [72]. Copyright 2020 the Chemical Society of Japan

### 3.2. RT_12_ MMPs

**Figure 10.**
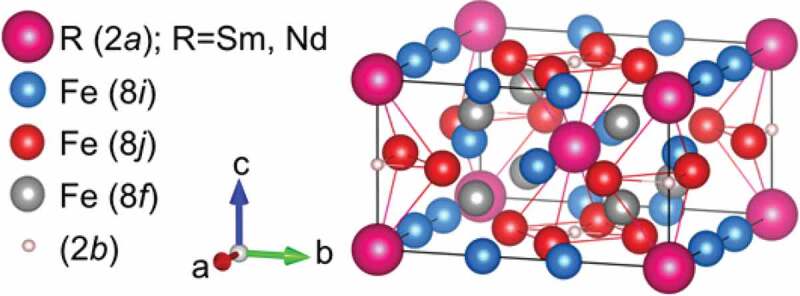
Crystal structure of RFe_12_ (ThMn_12_, *I*4/*mmm*) compounds (R = Sm, Nd)

NdFe_12_N (ThMn_12_-type, *I*4/*mmm*) and Sm(Fe,Co)_12_ (ThMn_12_-type, *I*4/*mmm*) compounds are the most impressive candidates for rare-earth-element-lean permanent magnets: their thin films were found to possess significantly large $M_{s}$ and $H_{a}$ (Figure 1), ${\left(BH\right)}_{max}$ theoretical limits of 550 and 630 $kJm^{-3}$, respectively, and high $T_{c}$ of 550 and 586°C, respectively; all the properties surpass those of the Nd_2_Fe_14_B [15,20,107–109]. Recently, anisotropic Sm(Fe_0.8_Co_0.2_)_12_–B films composed of columnar grains (40 nm) textured with amorphous B intergranular boundary have been realized. The films exhibited a large room-temperature $\mu_{0}H_{c}$ of 1.2 T, $\mu_{0}M_{r}$ of 1.5 T, and very small temperature-dependent $H_{c}$, promising excellent stability at *T* ≥ 150°C [110]. Unfortunately, they are metastable phases and, thus could not be realized in bulk for the fabrication of PMs. To this end, one can partially substitute Fe with stabilizing elements M (M = Ti, V, Cr, Mn, Co, Mo, W, Al, Si, Ga) in preferential 8*f*, 8*i*, and 8*j* sites, depending on the stabilizing elements, for example, Ti, V, and Mo in 8*i*; Co and Si in 8 *f* and 8 *j*; Ga in 8 *j*, as shown inFigure 10 [12,13,17,18,40–50,106,111–117]. The substitution with a large concentration of M leads to a significant reduction in $M_{s}$, except for Co, as shown in Figure 1 for Ti. The Co substitution results in the $M_{s}$ enhancement based on the Slater-Pauling curve, which is elucidated by an increase in the majority-spin state density below the Fermi level [114,117]. An effective strategy that stabilizes the low M-substituted concentration compounds and rationally enhances $M_{s}$ is to substitute Sm or Nd with other R elements of smaller atomic number (e.g., Zr, Y) in preferential 2*a* sites (Figure 10), though it reduces $H_{a}$ for Zr, as shown in Figure 1 [16,21,22,117–126]. The substituted compounds have been found to be stable at even very high temperatures [127]. The stabilization by the substitution can be understood as a decrease of local mismatches in interatomic distances in the structure unfavorable for the orbital hybridization [121], resulting in low formation energies of the substituted compounds [114,126]. Interstitial nitrogen doping into 2*b* octahedral sites in Nd(Fe,M)_12_ (Figure 10) is of crucial importance to enhance $M_{s}$ and $H_{a}$; it could be explained as the hybridization between N-*p* (2*b*) and Fe-*d* (8*j*) gives raises in the magnetic moment and the crystal field parameter $r^{2}A_{2}^{0}$ [128,129].

**Figure 11.**
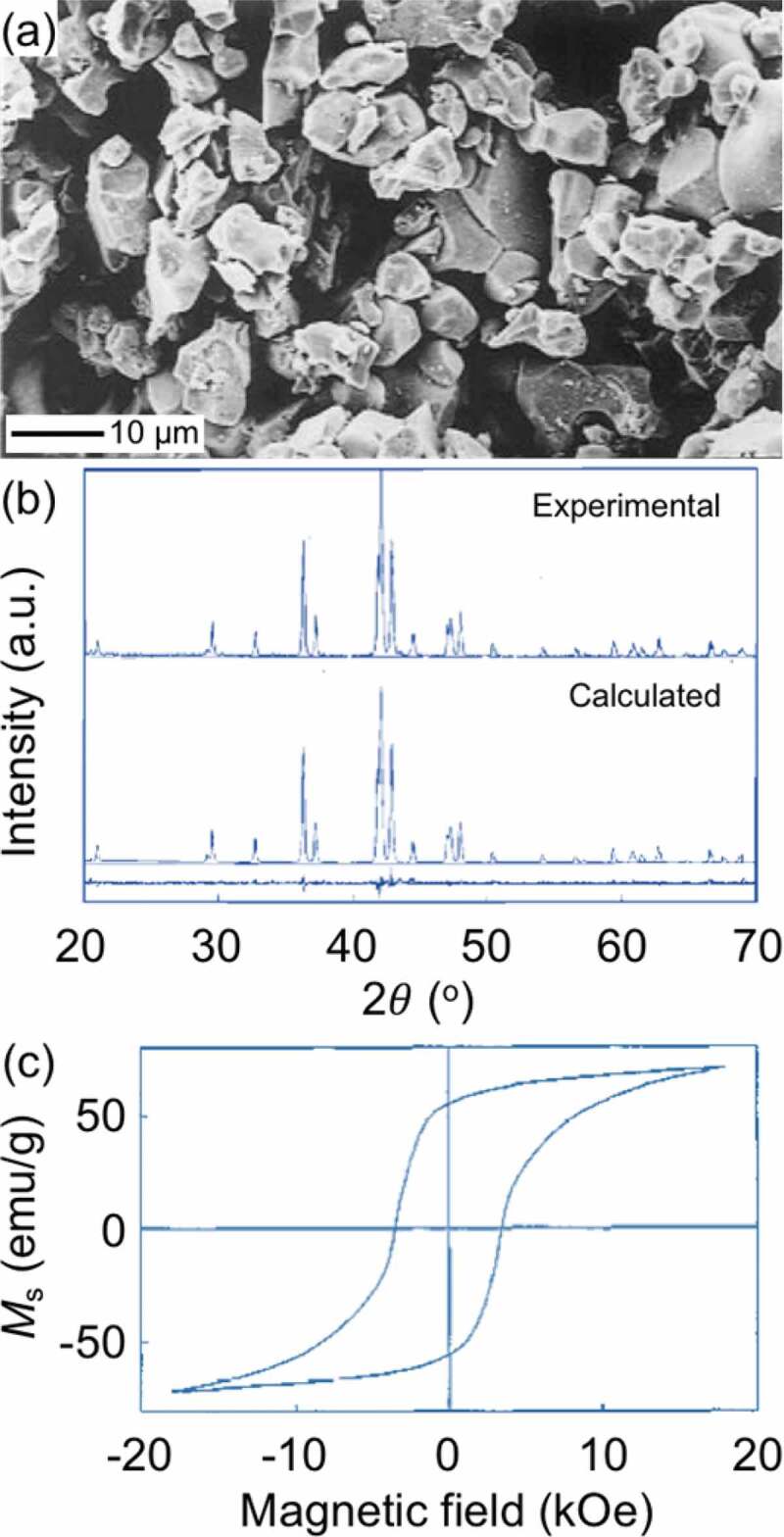
Structural analysis and room-temperature magnetic properties of chemically synthesized NdFe_10_Mo_2_ and NdFe_10_Mo_2_N MMPs (*I*4/*mmm*, 3–8 μm). (a) SEM image and (b) XRD patterns of NdFe_10_Mo_2_ MMPs synthesized using CaH_2_ R–D at 1010 °C for 4 h. (c) M-H curve of NdFe_10_Mo_2_N MMPs synthesized by nitridation of NdFe_10_Mo_2_ MMPs, showing $\mu_{0}H_{c}$ of 0.35 T. (a,b) Reproduced with permission from [98]. Copyright 2020 American Chemical Society. (c) Reprinted with permission from [131]. Copyright 2020 Elsevier

Despite such impressive $H_{a}$ and enormous research efforts, the RT_12_ MMPs with substantial $H_{c}$ have not been realized yet, the reported $H_{c}$ values being still less than 10% of the corresponding $H_{a}$ [40–50,111,112,124]. The following critical issues make it extremely difficult to practically realize the hard magnetic potential of RT_12_ compounds. Firstly, the size and shape of RT_12_ MMPs have not been well optimized, especially in the sub-micrometer range, as shown in Figure 2(b) [40–50,111,112,124]. Secondly, since equilibrium phases formed along with the typical RT_12_ phase are ferromagnetic, the conventional methods cannot introduce appropriate non-magnetic intergranular boundary phases, unlike Nd_2_Fe_14_B where their intergranular boundary is greatly facilitated by the eutectic reaction with Nd phase [8,21,111,130]. Finally, the RT_12_ compounds usually melt at relatively high temperatures, making it difficult to fabricate anisotropic magnets through the liquid-phase sintering [8,21,111,130]. Chemically synthesized fine RT_12_ MMPs with optimum microstructure may become suitable for sintering into fully dense anisotropic magnets, but is greatly challenging to be obtained. In a typical synthesis, a mixture of Fe_2_O_3_ (*P*4_1_2_1_2, *R*-3*c*), NdFeO_3_ (*Pnma*), and Fe_2_(MoO_4_)_3_ (*P*2_1_/*c, Pbcn*) NPs (*ca*. 200 nm), as described in Section 2 (Figure 6(t)), reduced by H_2_ at 700°C to convert into Fe(Mo) alloyed (*Im*-3*m*) and Nd_2_O_3_ (*C*2/*m, P*-3*m*1) NPs, and subsequently converted into NdFe_10_Mo_2_ (*I*4/*mmm*) MMPs (*ca*. 3–8 μm) by CaH_2_ R–D at 1010°C for 4 h (Figure 11(a,b)) [98]. The particle size of the oxide NPs was crucial in controlling the particle size of NdFe_10_Mo_2_ MMPs: the CaH_2_ R–D reaction could be carried out at lower temperatures (*e.g*., 960°C) and for a short time for the smaller oxide NPs (*ca*. 50 nm), resulting in smaller NdFe_10_Mo_2_ MMPs [98,131]. The nitridation of NdFe_11_Ti was conducted using the same procedure for the synthesis of Sm_2_Fe_17_N_3_, as described in Section 3.1, but at higher temperatures (550–600°C). As a result, the NdFe_10_Mo_2_N MMPs (*ca*. 3–8 μm) exhibited a room-temperature $\mu_{0}H_{c}$ of 0.35 T [131]. The method has been successfully applied to synthesize a series of ternary, quaternary, and quinary R(Fe,M)_12_X (R = Nd, Y, Nb, Tb, Er; M = Ti, Mo, W, Si; X = N, H) MMPs, as shown in Table 1 [131–136]. For the formation of the R(Fe,M)_12_X phase almost free from impurities such as Fe and/or TiFe_2_ phases, these syntheses were required to compensate the Sm evaporation in Ca R–D process of 2–10 at.% exceeded to the 1:12 stoichiometry, depending on R elements. Since the formation of R(Fe,M)_12_X phase required very high temperature (960–1100°C) for long reaction time (4–8 h), the particle sizes of resultant MMPs were in the range of 3–8 μm. The size control through thermodynamics, which is applicable for Sm_2_Fe_17_N_3_, is no longer effective for RT_12_ in the mesoscopic scale in such high-temperature reactions. The particle size may be further controlled to some extent through kinetics, in which the mixed oxide NPs are embedded in dispersant matrixes (e.g., CaO, GO) to suppress the resultant MMPs from sintering. A more effective approach to the kinetic control is to adopt encapsulated nanostructures as precursors (Figure 6(a–s)), where all elements are incorporated in a single NPs with homogeneous composition. We have examined the feasibility of this approach for the synthesis of quinary (Sm,Zr)(Fe,Co,Ti)_12_ MMPs using Sm–O, Zr–O, and Ti–O encapsulated CoFe_2_O_4_ (*Fd*-3*m*) NPs (5 nm), which was mentioned in Section 2. As a result, we have successfully synthesized ultrafine (Sm,Zr)(Fe,Co,Ti)_12_ MMPs with partially controllable size. The resulting (Sm,Zr)(Fe,Co,Ti)_12_ MMPs exhibited a relatively large room-temperature $H_{c}$ and high susceptibility to magnetic-field alignment, resulting in anisotropic bulk magnets with a large ${\left(BH\right)}_{max}$, which will be reported in the forthcoming paper. Taking these potentials into consideration, synthetic prospects will move a step forward in the size control through kinetics, which is advanced by the use of encapsulated nanostructure as precursors, to obtain their particle size in the range below 1 μm.

### 3.3. Oxidation and Hydrogenation of R–T MMPs

The chemical synthesis of R–T MMPs involves calciothermic reduction, resulting in the formation of byproducts (e.g., CaO, CaCO_3_) and residual reductants (e.g., CaH_2_ or Ca). Therefore, a post purifying process is required; however, it is still a challenge to achieve high purity and improved magnetic properties of R–T MMPs. The purification using water-based solutions as a rinsing agent has been found to form side products and, thus affect the magnetic properties of as-synthesized R–T MMPs to some extent [81]. Generally, water-rinsing induced oxidation and hydrogenation of R–T MMPs are inevitable side reactions. Owing to low ionization potentials, R elements can be easily oxidized to form surface oxides once R–T MMPs are exposed to air environment, especially in acidic solutions, leading to an attenuation of magnetic performance. Recently, a new washing process has been reported for highly effective purification and stabilization against oxidation of Sm–Co and Nd_2_Fe_14_B MMPs by Choa and co-workers [81]. In this procedure, a methanol solution of NH_4_Cl was used as a rinsing agent, in which Ca and CaO react with NH_4_Cl to form NH_3_ and methanol-soluble CaCl_2_. As a result, neither surface oxidation nor any damage to the MMPs was observed by HRTEM characterization, resulting in a substantial improvement of $M_{s}$, even near a $M_{s}$ theoretical value for Sm–Co MMPs. For the nanoscale R–T MMPs sensitive to air, the situation is much severe at elevated temperatures. Their protection against long-term and severe oxidation is crucially important for practical applications, especially for high-temperature applications. Recently, Sun et al. demonstrated a new strategy for the chemical synthesis and stabilization of SmCo_5_ NPs for high-performance nanomagnet applications in a broad temperature range [75]. In particular, the chemically synthesized SmCo–O NPs composed of Sm_2_O_3_ and CoO were coated with a layer of NGC (Figure 6(s)), embedded in CaO matrix, and reduced by Ca at 850°C to produce SmCo_5_@NGC MMPs. The resultant SmCo_5_@NGC MMPs showed efficient stability against oxidation: they could maintain 99.2% or 98.3% of magnetization after their exposure to air at room temperature for 5 days or 100°C for 48 h, respectively. The protection of R–T MMPs against oxidation is also necessary to prevent the oxidation induced decomposition. It has been found that the (Sm_0.8_Zr_0.2_)(Fe_0.75_Co_0.25_)_11.5_Ti_0.5_ phase was stable at very high temperatures up to at least 1100°C in an almost oxygen-free atmosphere, but was decomposed above 427°C [127].

Hydrogenation of R–T MMPs through the exothermic reaction of residual Ca with H_2_O in a rinsing process using water-based solutions has been found to considerably reduce $H_{c}$ of Sm_2_Fe_17_N_3_ MMPs [72,95,96]. As seen in Figure 9, the $H_{c}$ of Sm_2_Fe_17_N_3_ MMPs rinsed with distilled H_2_O is reduced by 40–80% those of as-synthesized ones. This could be understood as H atoms interstitially occupy available 18*g* tetrahedral sites (Figure 7) to cause a decrease in $H_{a}$ [137,138]. It was also obviously observed that the dehydrogenation of hydrogen-doped Sm_2_Fe_17_N_3_ MMPs by means of annealing in vacuum was ineffective to recover their $H_{c}$ [95]. As a result, the Sm_2_Fe_17_N_3_ MMPs (0.6 μm) exhibited room-temperature $\mu_{0}H_{c}$ of 2.8 T, 1.56 T, and 2.28 T in the form of anisotropic samples for as-synthesis, rinse with distilled water, and a combination of the rinse and dehydrogenation in a reduced atmosphere at 200°C, respectively. Slow oxidation of residual Ca to CaO, prior to the rising process, was then applied to avoid the *in situ* formation of H_2_, resulting in the $H_{c}$ preservation of as-synthesized Sm_2_Fe_17_N_3_ MMPs [96]. However, the oxidation also generated water-insoluble Sm_2_O_3_ and CaCO_3_ impurities, deteriorating $M_{s}$. The purification process using a NH_4_Cl/methanol solution, as previously described [81], is greatly capable of selectively rinsing out impurities and avoiding the *in situ* generation H_2_; it has been extended to successfully prepare dispersible Sm_2_Fe_17_N_3_ MMP [74]. Unlike what was observed in the Sm_2_Fe_17_N_3_ compound, hydrogen interstitial doping in preferential 2*b* octahedral sites in RT_12_ compound (Figure 10) has been found to enhance their $M_{s}$, $H_{a}$, and $T_{c}$ [132–135,139–147]. The increase in $H_{a}$ was attributed to an increase in the crystal field and a change in the local symmetry of 4*f*-electron shell along the *c*-axis, while the increases in $M_{s}$ and $T_{c}$ were attributed to an unit-cell-volume expansion and strong Fe–Fe exchange interactions, respectively, beyond the hydrogen interstitial doping [139–147].

## 4. R–T/T exchange-coupled NCMs

Magnetically hard/soft exchange-coupled NCMs have long been a potential candidate for high-performance permanent magnets since it can possess a large ${\left(BH\right)}_{max}$, which is deduced from large $H_{a}$ and $M_{s}$ of the corresponding hard and soft magnetic constituents, respectively [148,149]. Subject to the R elements crisis, they have the high potential to meet current demands for R-element-lean PMs with large ${\left(BH\right)}_{max}$ and operating temperatures of 150–200°C. The effectiveness of exchange-coupled interaction, which is represented by the microstructure factor α [25–29], is inversely proportional to the ratio $D_{s}/\delta_{w}$, where $D_{s}$ is the size of a soft magnetic phase and $\delta_{w}=\pi \sqrt{A_{ex}/K_{1}}$ is the domain wall width of a hard magnetic phase [148,149]. An effective exchange-coupled interaction requires the size of the soft magnetic phase being small enough ($D_{s}<2\delta_{w}$ for anisotropic bulk NCMs) and the size of the hard magnetic phase approaching the exchange length ($l_{ex}$) of the soft magnetic phase [148–151]. These fundamentals have led to two following approaches to the development in the exchange-coupled NCMs based on the hard magnetic materials. One is to adopt materials with a large $K_{1}$ but a consequently small $\delta_{w}$, such as *L*1_0_-FePt [152], Nd_2_Fe_14_B [153,154], SmCo_5_ [87,155–158], and Sm_2_Fe_17_N_3_ [150], as their large $H_{a}$ compensated for a small $M_{s}$ corresponding to a small $D_{s}$ ($D_{s}$ < *ca.* 10 nm) [148,149]. Another is to adopt materials with a moderate $K_{1}$ and a consequently relatively large $\delta_{w}$, such as *L*1_0_-FePd [159–161] and HfCo_7_ [162], as they had benefitted to α and $D_{s}$, and a large $M_{s}$ corresponding to a large $D_{s}$ ($D_{s}$ < *ca.* 20 nm) could be compensated for a moderate $H_{a}$ [148,149,161,162]. The latter approach is not usually considered, since the gain in α is overcompensated by the reduction of $H_{a}$.

Apart from the microstructure issues above, the most obvious obstacle to the synthesis of R–T/T NCMs is the formation of undesirable phases as the R and T metals can form many equilibrium and metastable phases. The design of magnetically hard/soft exchange-coupled NCMs with the soft phase as an equilibrium phase of the hard phase, which is similar to the one that was proved to be so effective in *L*1_0_-FePd/Fe NCMs [159–161], is a solution. Chemical synthesis of R–T/T NCMs has had very limited success in obtaining large ${\left(BH\right)}_{max}$. The Sm_2_Fe_17_N_3_ has a large $K_{1}$ of 16.2 $MJm^{-3}$ [19]; the Sm_2_Fe_17_N_3_ (2.4 nm)/Fe_65_Co_35_ (9 nm) alternatively multilayered anisotropic NCMs have been theoretically predicted to have a grant ${\left(BH\right)}_{max}$ of 1 $MJm^{-3}$ (120 MG Oe) [149]. Nevertheless, large ${\left(BH\right)}_{max}$ of Sm_2_Fe_17_N_3_ NCMs has never been realized, because there has still no their bulk materials prepared with a grain size of the order of 10 nm, where large $H_{c}$ and $M_{r}$ could be achieved in their NCMs [150,151]. Since this size range is an unrealistic practice for metastable Sm_2_Fe_17_N_3_, little attention has been paid to the Sm_2_Fe_17_N_3_ NCMs so far. Owing to it’s a huge $K_{1}$ of 17.2 $MJm^{-3}$, a relatively small $\mu_{0}M_{s}$ of 1.07 T [36], and easy chemical synthesis among R–T intermetallics, the SmCo_5_ has drawn considerable attention as a hard magnetic phase [87,155–158]. Generally, the SmCo_5_/Fe NCMs were synthesized by a simultaneous Ca R–D of mixed oxide NPs [87,155,156]; they exhibited very small $M_{r}$, resulting in very small ${\left(BH\right)}_{max}$. The main reason for such the reported small ${\left(BH\right)}_{max}$ in the SmCo_5_/Fe NCMs is a lack of an easy-axis alignment of the hard magnetic SmCo_5_ phase. It has been demonstrated that the anisotropic FePt/Fe_0.8_Ni_0.2_ NCMs could enhance ${\left(BH\right)}_{max}$ by 224% of that of the corresponding isotropic NCMs [163]. Therefore, chemical synthesis of particulate NCMs, which is similar to the ones that were proved to be so effective in *L*1_0_-FePd/Fe NCMs [161] and HfCo_7_/Fe_65_Co_35_ NCMs [162], is of crucial importance to fabricate the anisotropic NCMs with ultra-large ${\left(BH\right)}_{max}$. In a typical synthesis [157], particulate SmCo_5_/Co MMPs (200 nm, Co-soft phase: 4 wt.%) were synthesized by Ca R–D of Sm[Co(CN)_6_]⋅4H_2_O@GO MMPs and Co(acac)_2_ additive at 960°C; their isotropic sample showed room-temperature $\mu_{0}H_{c}$ of 2.07 T, $M_{r}/M_{s}$ of 0.75, and ${\left(BH\right)}_{max}$ of 80 $kJm^{-3}$ (10 MG Oe). This small ${\left(BH\right)}_{max}$ resulted from a low fraction of the soft magnetic Co phase (4 wt.%) and/or too large grain size of the hard magnetic SmCo_5_ phase (*ca.* 200 nm) to an effective exchange coupling [148–151]. It is highly perspective to gain a drastically enhanced ${\left(BH\right)}_{max}$ as the particulate SmCo_5_/Co MMPs can be magnetically aligned to form the anisotropic NCMs. In the line with the problem of R–T/T NCMs, the great challenge in the synthesis of SmCo_5_/Fe(or Co) NCMs is to achieve a high fraction of the soft phase since other phases can be formed with the increase in the fraction [155,157]. An effective chemical synthesis avoiding this alloying issue is to disperse SiO_2_-coated Fe NPs in SmCo_5_ matrix, which is described in the literature elsewhere [156]. Prospects are presumably better for RT_12_/T NCMs, where T is the only equilibrium phase of the RT_12_ phase at their high fraction. There have unfortunately been no reports on the RT_12_/T NCMs yet.

## 5. Conclusions and prospects

Multicomponent R–T permanent magnetic materials, which are multielement or multi-phase ones, are expected to shape the growth of ${\left(BH\right)}_{max}$ back to the ‘Moore’s law’ after decades of Nd_2_Fe_14_B magnets since the theoretical ${\left(BH\right)}_{max}$ limit is given by $\mu_{0}M_{s}^{2}/4$ while the $M_{s}$ can further increase (Figure 1). However, extraordinary achievements in searching for high-performance magnetic materials with ultra-large intrinsic properties with the aid of theoretical calculations in recent years, especially (R,Zr)(Fe,Co,Ti)_12_ (R = Nd, Sm) compounds (Figure 1), leave a large gap behind them to the fabrication of their corresponding magnets. This lag practically comes from challenges in the synthesis of these compounds with optimum microstructure in the mesoscopic range for maximizing practically relevant extrinsic properties and the introduction of appropriate grain boundaries for the fabrication of their anisotropic magnets. While conventional synthetic techniques that are popular with the production of magnetic material powders remain unsatisfactory, chemical synthetic approach with recent advances in the solution-phase synthesis of nanostructured precursors followed by solid-state reaction may become suitable to overcome the challenges. As nanostructured precursors are used, the chemical approach broadens microstructural control horizons given by thermodynamics and kinetics. The thermodynamics, unfortunately, addresses a limit in the size control of RT_12_ intermetallics, where their formation requires very high temperatures. Prospects seem to be only adopting the kinetic control, where appropriate engineering of nanostructured precursors toward the following solid-state reaction is a crucial key. The ideal nanostructured precursors are particulate NPs composed of all constituents, and they are monodisperse and homogenous in composition. In addition to the precedent challenges in the synthesis of R–T intermetallics, the synthesis of R–T/T NCMs faces new problems of achieving the R–T phase with a particle size of the order of 10 nm for effective exchange interactions, and the formation of undesired phases with an increase in the T fraction. As a result, while monodisperse Sm_2_Fe_17_N_3_ MMPs with very well-controllable size and ultra-large $H_{c}$ are ready for the fabrication of anisotropic magnets, progress in the development of (R,Zr)(Fe,Co,Ti)_12_ MMPs and R–T/T NCMs remains marginal. Once MMPs with primarily optimum microstructure are synthesized, their anisotropic magnets can be fabricated by a rapid low-temperature current sintering method [164] or an infiltration treatment [165], instead of conventional liquid-phase sintering.
